# Conservative management of uterine adenosarcoma: lessons learned from 20 years of follow-up

**DOI:** 10.1007/s00404-019-05306-6

**Published:** 2019-10-04

**Authors:** Ariadne L’Heveder, Benjamin P. Jones, Srdjan Saso, Jen Barcroft, Robert Richardson, Baljeet Kaur, Sadaf Ghaem-Maghami, Joseph Yazbek, J. Richard Smith

**Affiliations:** 1 West London Gynaecological Cancer Centre, Hammersmith Hospital, Imperial College NHS Trust, London, London, W12 OHS UK; 2 Department of Surgery and Cancer, Imperial College London, Du Cane Road, London, W12 0NN UK; 3 Department of Gynaecology, Chelsea and Westminster Hospital, 369 Fulham Road, Chelsea, London, SW10 9NH UK; 4 Department of Histopathology, Imperial College London, Du Cane Road, London, W12 0NN UK

**Keywords:** Gynaecology, Oncology, Surgery, Hysteroscopy, Adenosarcoma of the uterus, Fertility preservation

## Abstract

**Purpose:**

Uterine adenosarcomas (UAs) account for 5–8% of cases of uterine sarcomas. Treatment includes total abdominal hysterectomy (TAH) and bilateral salpingo-oophorectomy (BSO).

Fertility preservation is an emerging concept in gynaecology oncology and is particularly relevant in UA, where cases are diagnosed as young as 15-year-old. This manuscript demonstrates a case of UA which was treated conservatively, achieved successful livebirths and underwent completion hysterectomy after two decades of follow-up.

**Method:**

This was a retrospective case note review.

**Results:**

An 18-year-old nulliparous woman presented with abnormal vaginal bleeding. Ultrasound identified an endometrial polyp, which was histologically diagnosed as low-grade adenosarcoma. She was advised to undergo TAH and BSO, but instead decided to preserve her fertility and opted for conservative management. She was monitored with pelvic ultrasound, hysteroscopy and endometrial biopsy bi-annually, with annual pelvic magnetic resonance imaging for 10 years which was uneventful. 11 years post-operatively she conceived following in-vitro fertilization (IVF) but suffered a miscarriage at 16 weeks likely due to cervical incompetence. She subsequently conceived with twins. She delivered spontaneously preterm at 28 weeks. Both children are alive and well. After 20 years of follow-up, she underwent a laparoscopic hysterectomy with no evidence of recurrence. She remains disease free.

**Conclusion:**

Whilst radical completion surgery should be advised in UA, this case, in addition to all published conservatively managed cases of UA, demonstrates that conservative management is possible in appropriately selected women. Intensive monitoring post-operatively is essential owing to the risk of recurrence; however, this may pose deleterious side effects which require consideration.

## Introduction

Uterine adenosarcoma (UA) accounts for 5–8% of cases of uterine sarcomas, a rare form of neoplasm comprising less than 10% of uterine malignancies [1]. Whilst leiomyosarcomas remain the most common uterine sarcomas, other subtypes include endometrial stromal sarcomas, undifferentiated endometrial sarcomas and adenosarcomas [2]. The staging system for uterine sarcomas was revised in 2009 by the International Federation of Gynaecology and Obstetrics (FIGO) when it was considered distinct from endometrial carcinoma [3].

Adenosarcomas are typically defined by the presence of a benign appearing epithelial component in combination with a low-grade sarcomatous component often resembling endometrial stromal sarcoma [4]. Carcinosarcomas differ from UA owing to the presence of malignant epithelium [5]. As such, UAs are considered to be less aggressive and associated with a more favourable prognosis than their high-grade counterpart, carcinosarcomas [6]. However, UA with sarcomatous overgrowth (SO), defined as the presence of pure sarcoma occupying at least 25% of the tumour [2], is associated with worse outcome and higher risk of recurrence [2, 3, 5, 7–9], with malignant potential comparable to high-grade sarcomas [10]. Myometrial invasion (MI), heterologous elements, lymphovascular space invasion and advanced stage have also been associated with a worse prognosis [11].

UA most commonly presents with vaginal bleeding, but may cause pelvic pain, vaginal discharge, and symptoms related to uterine enlargement [5, 7, 8, 12]. Primary treatment traditionally includes hysterectomy and bilateral salpingo-oophorectomy (BSO) [13]. The role of lymph node dissection (LND) and adjuvant chemo-radiotherapy remains unclear [1, 4]. The percentage of cases with lymph node involvement is between 0 and 6% [11]; therefore, LND may be unnecessary in women with disease confined to the uterus. However, in those with bulky disease, LND should be considered [4, 14]. In women with stage I disease with SO, adjuvant therapy has been demonstrated to be associated with better overall and progression-free survival [11], albeit not to a significant extent. Moreover, adjuvant therapy may reduce recurrence in women with SO and/or MI [4], although other data are conflicting [6].

Fertility preservation is an emerging concept within gynaecological oncology. This is secondary to the fact that up to 10% of invasive cancers occur in women aged under 45 [15]. Fertility sparing surgery (FSS) necessitates an approach that balances the obligation to remove pathology safely but preserves the essential components for reproduction. It has been made possible by earlier diagnosis and treatment, as a result of greater patient awareness and earlier presentation. This is in addition to enhanced screening, more accurate diagnostic capabilities and the evolution of surgical techniques [16].

With the rising age of motherhood globally [17], the demand for fertility preservation, including FSS, is likely to increase simultaneously in the future. This is particularly relevant in the context of UA given the younger age at presentation compared to the more prevalent endometrial tumours [4], with diagnosis as young as 15 [11]. Moreover, given that UA is generally considered low grade with an indolent course, there may be a role for FSS in carefully selected cases.

We present a case of a young woman with UA who was treated conservatively, subsequently achieved livebirth and finally underwent completion hysterectomy after two decades of follow-up.

## Case report

An 18-year-old nulliparous woman presented with abnormal vaginal bleeding. Ultrasound detected the presence of an endometrial polyp, which was confirmed on hysteroscopy and removed using a resectoscope. The polyp was approximately 2 cm in length. Histological analysis revealed benign endometrial glands with moderately cellular stromatolites showing some degree of periglandular condensation and mild pleomorphism. No SO or MI was noted. The histological diagnosis of low-grade adenosarcoma was made. Subsequently, the patient was advised to undergo hysterectomy, BSO and lymphadenectomy, but she declined to preserve her fertility. She was referred for a second opinion and following extensive counselling, opted for conservative management.

Over the following 10 years, she was monitored with pelvic ultrasound, hysteroscopy and endometrial biopsy every 6 months, and with annual pelvic magnetic resonance imaging. Follow-up was largely uneventful, with normal imaging and histology. Nine years post-operatively an abnormal appearing area on the posterior aspect of the lower uterine segment was identified on hysteroscopy and subsequently resected. Histology was benign with no features of recurrence.

Eleven years post-operatively, she underwent in vitro fertilization (IVF) and successfully conceived following the first cycle. Unfortunately, she suffered a second trimester miscarriage at 16 weeks’ gestation, with a history suggestive of cervical incompetence after admission with a dilated cervix and bulging membranes. The following year she conceived twins following another IVF cycle. She underwent an uncomplicated elective trans-vaginal cervical suture. She delivered male twins at 28 weeks’ gestation following pre-term labour, which was attributed to placental abruption. Both children are alive and well. She was offered hysterectomy after delivery, and at each subsequent follow-up appointment, but declined as she was uncertain as to whether or not her family was complete. Following pregnancy, her follow-up intensity was reduced to annually with continued ultrasound scans along with hysteroscopy and endometrial biopsy. She continued to defer definitive treatment until, after a total of 20 years of follow-up, she finally underwent an uncomplicated laparoscopic hysterectomy with ovarian conservation. There was no histological evidence of disease recurrence.

## Discussion

This case demonstrates the feasibility of FSS in appropriately selected women with UA. Conservative management of adenosarcoma is rare, with only ten previously published cases, as summarised in Table 1. All women who underwent uterine-preserving surgery were categorised as FIGO stage I. Six women (60%) were treated with hysteroscopic resection, whilst the remainder (*n* = 4; 40%) underwent dilatation and curettage (D&C) ± polypectomy. Two (20%) women received adjuvant chemotherapy: one had nine cycles of vincristine, dactinomycin and cyclophosphamide following polypectomy with no residual disease at a subsequent D&C [11], whilst the other was treated with ifosfamide and cisplatin [9]. Follow-up ranged from 12 to 132 months with a mean of 57.6 months (SD 44.5 months). Six women (60%) were disease free at final follow-up. Two (20%) had persistent disease confined to the uterus on imaging which was re-treated with D&C, with no further follow-up described thereafter [9]. Three (30%) women suffered recurrence. One woman who recurred had SO at initial diagnosis and recurred with peritoneal seeding 10 months following treatment with adjuvant chemotherapy. She subsequently underwent total abdominal hysterectomy (TAH) and BSO, pelvic LND, lower anterior resection, and tumour excision. She was alive and disease free at follow-up almost 2 years later [9]. Another woman recurred 8 years following primary treatment, after she had given birth, when an area of thickened endometrium was seen on ultrasonographic monitoring; low-grade recurrence was confirmed histologically. She underwent radical completion surgery including TAH, BSO and LND and was disease free at final follow-up 4 years later. The final recurrence was identified as a suspicious mass in the endometrial cavity on ultrasound. However, she declined further hysteroscopy, thereby preventing histological diagnosis, and also declined further surgical intervention [9]. All ten women were alive at final follow-up. Two pregnancies were achieved both resulting in live births [9, 18, 19]. One delivered vaginally at 39 + 2 weeks’ gestation, 17 months after local excision of her UA [9]. The other had a vaginal delivery at term 3 years post-diagnosis [19]. She eventually recurred and underwent radical completion surgery as described previously [19].

**Table 1:** Previously reported cases of uterine adenosarcoma managed with uterine preservation techniques

Authors	Number of cases (*n*)	Age of patient (years)	FIGO stage	Sarcomatous overgrowth	Primary treatment	Adjuvant/post op-operative therapy	Progression free survival (months)	Recurrence	Further surgery	Status at last follow-up	Livebirth	Death
Bernard et al. [4]	1	NS	NS	N	D&C	NS	NS	N	N	Disease free	NS	N
Carroll et al. [11]	1	15	1	N	D&C + PP	V/D/C	132	N	N	Disease free	NS	N
Lee et al. [9]	7	33	IB	N	Transcervical resection	N	12	N	N	Disease free	N	N
		33	IA	N	Transcervical resection	N	77	N	N	Disease free	Y	N
		40	IA	N	D&C	N	32	N	N	Disease free	N	N
		21	IB	N	Transcervical resection	N	NS	N	D&C	Persistent disease	N	N
		22	IA	N	Transcervical resection	N	NS	N	D&C	Persistent disease	N	N
		27	IA	Y	Transcervical resection	I/P	27	Y	TAH + BSO + LND, lower AR	Persistent disease with recurrence	N	N
		27	IB	N	Transcervical resection	N	13	Y	N	Persistent disease with recurrence	N	N
Goh et al. [19]	1	21	I	NS	D&C + PP	N	96	Y	TLH + BSO + LND	Disease free	Y	N

*AR* anterior resection, *CT* chemotherapy, *D&C* dilatation and curettage, *FIGO* International Federation of Gynecology and Obstetrics, *I/P* ifosfamide/cisplatin, *LVSI* lymphovascular space invasion, *MPA* medroxyprogesterone acetate, *N* no, *NS* not specified, *PP* polypectomy, *TAH* total abdominal hysterectomy, *TLH* total laparoscopic hysterectomy, *V/D/C* vincristine/dactinomycin/cyclophosphamide, *Y* yes

Whilst there are limited data on recurrence following conservative treatment in UA, previous studies have demonstrated that women can remain disease free for beyond 10 years following FFS [11]. Conversely, however, disease may recur within a year [9]. Risk of recurrence increases with stage of disease, increasing age and the presence of SO and/or MI [4, 5, 14]. Recurrence rates following UA managed with hysterectomy + BSO range from 14% for low-grade disease without SO [18], to 70–80% in cases with SO [11], with an average of 26–40% [4, 11]. The overall recurrence rate in previously published FSS cases was 30% (*n* = 3). One patient had stage IA disease with SO whilst the second had stage IB disease [9]. The other recurrence was in a woman with unspecified stage I disease, with uncertain SO or MI status. No recurrences were noted in cases confirmed to have stage IA disease, without SO. This highlights the need for appropriate patient selection and reaffirms that FSS can be considered in women with stage IA disease without SO.

In conservatively managed cases, intensive monitoring and follow-up post-operatively are essential [3, 4]. Limited detail of the surveillance methods utilised in previous cases can be found. The use of ultrasound [13], and endometrial sampling [11, 19], has been described with successful detection of recurrence in an asymptomatic woman [19]. Given that UAs can recur after many years, prolonged surveillance is necessary [4].

An important consideration is the potentially deleterious physical and psychological effects of increased surveillance. The management described herein utilised multiple hysteroscopies and endometrial biopsies, initially every 6 months, then annually from 10 years onwards. This resulted in over 20 hysteroscopies throughout an 11-year period between index operation and first pregnancy, which resulted in a late miscarriage at 16 weeks’ gestation. Whilst no known association between multiple hysteroscopies and cervical incompetence can be found, it is highly likely that this contributed. A previous systematic review on miscarriage did not identify hysteroscopy as a risk factor [20], although it is likely that this is based on small numbers of hysteroscopies. Therefore, it is not appropriate to extrapolate this into the context of a woman who had 15–20 hysteroscopies pre-conception. Another consideration is whether the placental abruption which resulted in the premature birth of the woman’s twins was related to the follow-up regimen. Placental abruption has been reported to be more likely in cervical cerclage [21], multiple gestation pregnancies and following assisted conception [22], all of which were present in this case. Furthermore, placental abruption has been suggested to occur following damage to the endometrial lining after curettage which may result in abnormal placentation [23]. It is possible that the multiple endometrial biopsies which were taken during the monitoring in this case may have been a contributing factor.

The final consideration is the need for completion surgery once the woman’s family is complete. Along with a hysterectomy, traditional management of UA includes BSO to exclude and treat ovarian involvement [4, 24]. Furthermore, as most subtypes of uterine sarcoma present oestrogen and progesterone receptors, BSO reduces potential endogenous sex hormone stimulated risk of recurrence [5, 18, 24]. Conversely, given that completion surgery will still be undertaken during reproductive years, ovarian preservation avoids primary ovarian insufficiency (POI) and its associated complications and negative impact on quality of life [25]. The case we present herein was counselled on the risks and benefits of BSO versus ovarian preservation but, as she was 38 years old at the time, decided to preserve her ovaries to avoid POI. Various studies have demonstrated low rates of ovarian involvement in cases of UA [9, 14], concluding that ovarian preservation in women of reproductive age is a reasonable option in the absence of metastasis macroscopically or on imaging. Moreover, retrospective data have demonstrated that ovarian preservation is not associated with worse oncological outcomes, with no difference in overall or cancer-specific survival [24]. However, caution is needed in the interpretation of these findings, owing to the low number of cases involved.

## Conclusion

**Fig. 1:**
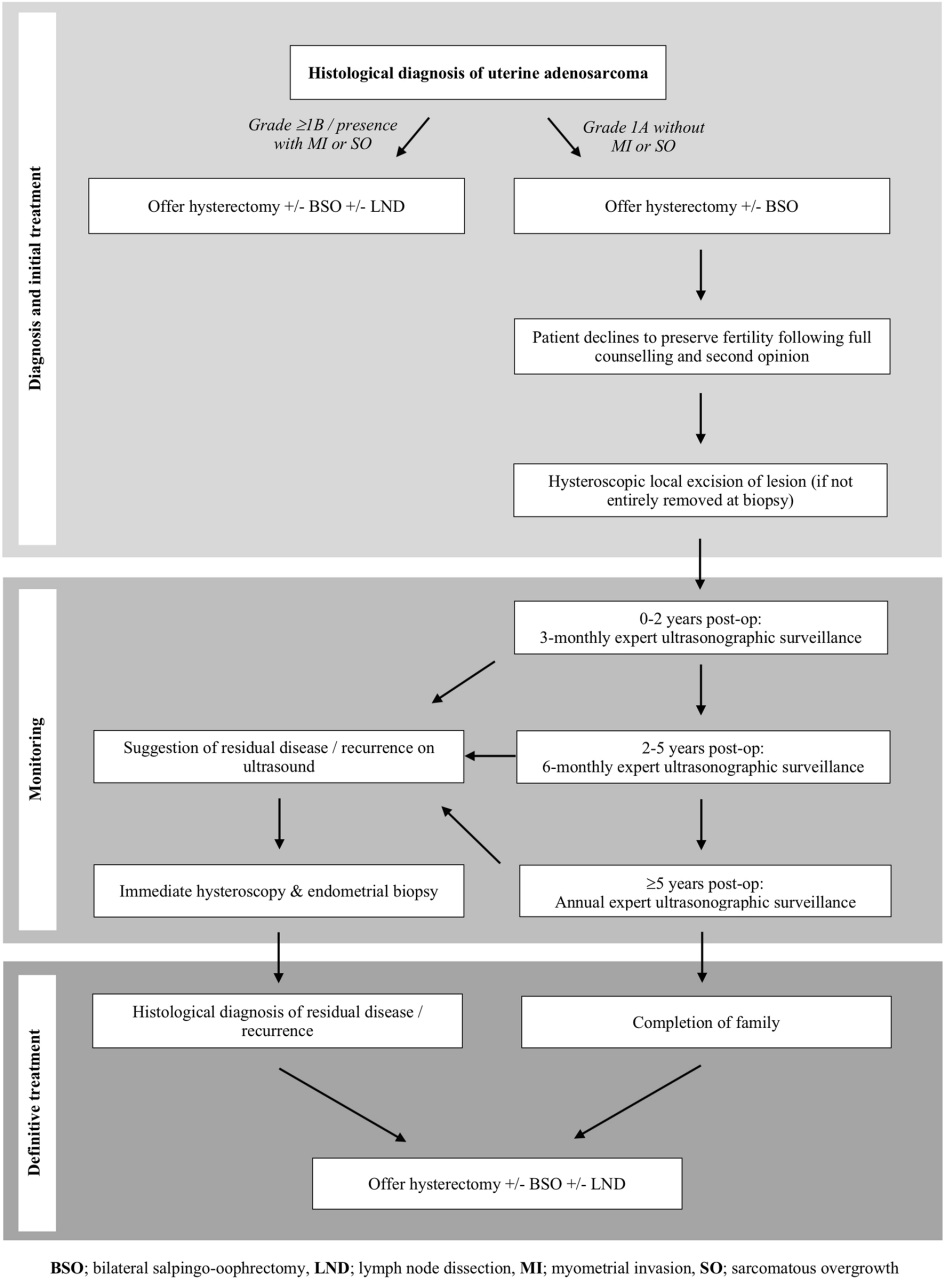
Proposed management algorithm for uterine adenosarcoma

In the context of rising maternal age and a preponderance to impact women of reproductive age, FSS may become increasingly requested in UA. We present herein a case which was treated with local excision. The patient subsequently went on to have a successful pregnancy and remained disease free for 20 years before undergoing completion surgery with ovarian preservation. Whilst we would advocate that all cases of UA should be advised to undergo hysterectomy ± BSO, conservative management can be considered in women of childbearing age who have not yet completed their families. Criteria for management would be a low-grade tumour and stage IA disease without SO or MI. A proposed management algorithm for UA is demonstrated in Fig. 1.

Post-operatively, expert ultrasonographic surveillance should be undertaken 3 monthly for the first 2 years, 6 monthly until 5 years post-operatively, and annually thereafter, in a similar fashion to other moderate to high-risk gynaecological cancers [26]. A low threshold for hysteroscopy and endometrial biopsy should be applied if an abnormality is identified on ultrasound. If circumstances allow, early conception should be recommended. Radical completion surgery including hysterectomy ± BSO should be advised following family completion. All women must be fully informed, including the risk of recurrence, and potential obstetric complications should multiple hysteroscopies and endometrial biopsies be required. Given the small numbers of reported cases treated conservatively so far, more cases with longer follow-up are necessary before FSS can be recommended, and optimal management and follow-up regimens can be defined.
